# EZH2 enhances expression of CCL5 to promote recruitment of macrophages and invasion in lung cancer

**DOI:** 10.1002/bab.1875

**Published:** 2020-04-16

**Authors:** Lilong Xia, Xinhai Zhu, Lei Zhang, Yanhui Xu, Guoping Chen, Jing Luo

**Affiliations:** ^1^ Department of Thoracic Surgery Zhejiang Hospital Xihu district Hangzhou Zhejiang 310000 China

**Keywords:** chemokines, CCL5, EZH2, lung cancer, macrophage recruitment, metastasis

## Abstract

EZH2 (enhancer of zeste homolog 2) regulates epigenetic gene silencing and functions as critical regulators in various tumor progression. Macrophages infiltration promotes cancer development via stimulating tumor cell migration and invasion. However, the effect of EZH2 on macrophages infiltration, cell invasion, and migration of lung cancer remains to be investigated. In this study, we found that knockdown of *EZH2* inhibited macrophages chemotaxis and decreased chemokine ligand 5 (CCL5). Wound‐healing and transwell assays results showed that migration and invasion of lung cancer cells was inhibited by *EZH2* deletion. Moreover, *EZH2* overexpression increased CCL5 expression. Loss‐of functional assay indicated that the promotion ability of EZH2 on macrophages chemotaxis was inhibited by *CCL5* knockdown. Mechanistically, the promotion ability of EZH2 on cell migration and invasion of lung cancer was also inhibited by *CCL5* knockdown. The *in vivo* subcutaneous xenotransplanted tumor model also revealed that silence of *EZH2* suppressed lung cancer metastasis and macrophages infiltration via regulation of CCL5. In conclusion, our findings indicated that EZH2 promoted lung cancer metastasis and macrophages infiltration via upregulation of CCL5, which might be the underlying mechanism of EZH2‐induced lung cancer cell progression.

## Graphical abstract

xxxx

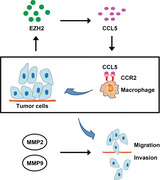

**Abbreviations**
ASCL1: Achaete‐scute family bHLH transcription factor 1
CCL5: chemokine ligand 5
DAB: diaminobenzidine
DKK1: Dickkopf‐1
EZH2: Enhancer of zeste homolog 2
FBS: fetal bovine serum
H3K27: tri‐methylation of histone H3 lysine 27
HE: Hematoxylin and eosin
IACUC: Institutional Animal Care and Use Committee
IHC: immunohistochemical
MMP: Matrix‐metalloproteinases
NSCLC: non‐small‐cell lung carcinom
PRC2: polycomb‐repressive complex 2
PVDF: polyvinylidene pluoride
RKIP: Raf kinase inhibitor protein
RPMI‐1640: Roswell Park Memorial Institute 1640
SDS‐PAGE: sulfate‐polyacrylamide gelelectrophoresis
TME: Tumor microenvironment

## 1 Introduction

Lung cancer is one of the most malignant tumors 1, with 80% of the incidence is non‐small‐cell lung carcinoma (NSCLC) 2. Owing to highly metastasis, the 5 year survive rate of advanced lung cancer is still below 5% and causes more mortality than other tumors 3. Therefore, effective treatment approaches for anti‐metastatic and anti‐invasion to lung cancer are desperately needed.

Tumor microenvironment (TME), composed of macrophages, fibroblasts, endothelial cells, and extracellular matrix and signal molecules, is a cell environment around tumor cells 4. The cancers cells acquired novel interdependencies with other cells (such as stromal and immune cells) in TME to facilitate the survival and even metastasis of the tumor cells 5. Among cells in TME, macrophages are one of the most common cells in shaping TME as well as providing conducive factors for tumor growth 6. Infiltration of macrophages associated with tumors is considered as one of the independent prognostic factors of NSCLC 7. Moreover, tumor cells also secret chemokines for macrophage recruitment and tumor growth 8. Among these chemokines, CCL5 has been proved to be one of the key chemotactic factors for macrophage recruitment in tumor cells 9, 10. Therefore, inhibition of macrophage recruitment via blockage of CCL represents one of the approaches to combat cancer progression 11.

EZH2 is a common histone–lysine methyltransferase and the enzymatic subunit of PRC2 (polycomb‐repressive complex 2) 12. EZH2 can catalyze mono‐, di‐, and trimethylation of histone H3 lysine 27 (H3K27) and participate in epigenetic gene silencing via regulating a chromatin structure 13. EZH2 was found to be overexpressed and performs oncogenic activity in many tumors 14. In lung cancer, EZH2 was upregulated and increased H3K27me3 to repress Dickkopf‐1 (Dkk‐1) expression 15. Knockdown of EZH2 inhibited cell proliferation of NSCLC via upregulation of p53 and upregulation modulator of apoptosis 16. Moreover, EZH2 promoted small cell lung cancer cell progression via TGF‐β‐Smad‐ASCL1 (Achaete‐scute family bHLH transcription factor 1) pathway 17. However, the functional role of EZH2 on macrophage recruitment in lung cancer remains to be investigated.

Therefore, we scoped to investigate the role of EZH2 in tumor cell behavior and in macrophage chemotaxis to evaluate the involvement of EZH2 in lung cancer. Our results showed that EZH2 can regulate lung cancer invasion and migration via inhibiting recruitment of macrophages. Our study enriched the molecule understanding for the metastasis of lung cancer and inspired a possible new strategy for preventing lung cancer.

## 2 Materials and methods

### 2.1 Cell culture

Human lung cancer cell lines‐A549 cells and A129L (Cell Bank of Type Culture Collection of Chinese Academy of Science; Shanghai, China) were maintained in Roswell Park Memorial Institute 1640 (RPMI‐1640, Gibco, Thermo Fisher Scientific, Waltham, MA, USA) containing 10% fetal bovine serum (FBS; Gibco) at 37 °C. Macrophage cell line RAW264.7 cells (American Type Culture Collection, Rockville, MD, USA) were cultured in Dulbecco's modified Eagle's medium with 10% FBS in 5% CO_2_ at 37 °C.

### 2.2 Cell transfection

pcDNA3.1‐EZH2 was obtained from AxyBio (Changsha, China) for the overexpression of EZH2. EZH2‐shRNA1# (:5′‐AAGCTAAGGCAGCTGTTTCAG‐3′) or 2# (5′‐ATCACTGTCTGTATCCTTTGA‐3′) and negative control (NC‐shRNA, 5′‐CCGGCAACAAGATGAAGAGCACCAACTCGAGTTGGTGCTCTTCATCTTGTTGTTTTT‐3′), CCL5‐siRNA (5′‐CCTCGCTGTCATCCTCATT‐3′), and siRNA negative control (NC‐siRNA, 5′‐AAUUCUCCGAACGUGUCACGU‐3′), were ordered from Guangzhou RiboBio (Guangzhou, China). The cells were seeded into 12‐well plates with 80% confluence and starved overnight and then were transfected with 300 ng pcDNA3.1‐EZH2 or pcDNA3.1, 100 nM EZH2‐shRNA1#, EZH2‐shRNA2#, NC‐shRNA, CCL5‐siRNA, or NC‐siRNA using Lipofectamine® 3000 (Thermo Fisher Scientific). After 48 H of transfection, the cells were collected for the following experiments.

### 2.3 Wound‐healing assay

Migration ability of lung cells was determined by undertaking wound‐healing assay 18. After 48 H of transfection, the cells were seeded into six‐well plate with 90% confluence. The monolayer cells were gently scratched using 200 µL Eppendorf tip. After removing the detached cells, the remained attached cells were incubated in culture medium without serum at 37 °C in a 5% CO_2_. After 24 H, cell migration was observed using a light microscope (400×) (Nikon N‐E; Nikon Corporation, Tokyo, Japan).

**Highlights**

- EZH2 enhanced the recruitment of macrophages to promote lung cancer progression via upregulation of CCL5
- Downregulation of EZH2 inhibited the *in vivo* macrophages infiltration of lung cancer via downregulation of CCL5

### 2.4 Transwell assay

A Matrigel‐precoated invasion chamber (Corning Costar, Cambridge, MA, USA) was used to detect the cell invasion 19. Cells at a density of 2 × 10^5^ cells/mL were plated to the upper chambers with 75% confluence and maintained in culture medium without FBS. The lower chamber contained culture medium with 15% FBS. During the incubation progress at 37 °C incubator, the cells would invade through the lower membrane and the cells without invasion ability would stay in the upper chamber. The cells that went through the basement membrane layer were fixed for 15 Min using methanol and stained with crystal violet (0.1%).

### 2.5 Chemotaxis assay

Macrophages (2 × 10^5^ cells/mL) were seeded to the upper chamber of 24‐well Transwell inserts (Corning Costar) with 80% confluence. The predescribed conditional medium with 10% FBS was, respectively, added to the lower chambers. The macrophages were incubated for 8 H at 37 °C. The collected macrophages migrated or invaded into the lower chamber were fixed and stained as described in the cell invasion assay. Five randomly fields under a light microscope (400×) were counted to analysis the cell invasion rate of macrophages.

### 2.6 Enzyme‐linked immunosorbent assay

The CCL2, CCL5, and CXCL2 protein concentration was detected by using the human CCL2/MCP‐1 quantikine enzyme‐linked immunosorbent assay (ELISA) kit from R&D system (Minneapolis, MN, USA), CCL5 Human Simple Step ELISA TM kit, and human MIP2 ELISA kit (CXCL2) from Abcam (Cambridge, MA, USA), respectively, according to the manufacturer instructions.

### 2.7 qRT‐PCR assay

Total RNA was isolated using Trizol reagent (Thermo, USA). M‐MLV reverse transcriptase, random hexamer primers, and dNTP (Promega, Madison, WI, USA) were used to synthesize cDNA according to the manufacturer's instructions. The LightCycler DNA Master SYBR Green I kit (Roche Diagnostics, Mannheim, Germany) was used to detect the mRNA expression on the LightCycler system (Roche Diagnostics). The 2^−ΔΔ^ *^CT^* method was used to analyze the relative expression to GAPDH. The primer sequences were as listed in Table 1.

**TABLE 1:** Primmer sequences

Gene	Sequence
EZH2	Forward: 5′‐ATTTCGTAGGAGGGAGCAAAG‐3′
	Reverse: 5′‐TGGGCCTGCTACTGTTATTG‐3′
CCL2	Forward: 5′‐ATCACCAGCAGCAAGTGTC‐3′
	Reverse: 5′‐AGGTGGTCCATGGAATCCTG‐3′
CCL5	Forward: 5′‐CCAGCAGTCGTCTTTGTCAC‐3′
	Reverse: 5′‐CTCTGGGTTGGCACACACTT‐3′
CXCL2	Forward: 5′‐AGTGGCAAATCCAACTGACC‐3′
	Reverse: 5′‐AAACACATTAGGCGCAATCC‐3′
GAPDH	Forward: 5′‐TGCACCACCAACTGCTTAGC‐3′
	Reverse: 5′‐GGCATGGACTGTGGTCATGAG‐3

### 2.8 Western blot assay

The isolated proteins (20 µg per lane) were separated using 10% sodium dodecyl sulfate‐polyacrylamide gelelectrophoresis (SDS–PAGE) and transferred onto polyvinylidene fluoride membrane (Millipore, Bedford, MA). The skimmed milk (5%) was incubated with the membranes at 37 °C for 2 H. The primary antibody, including anti‐EZH2 (Abcam, ab186006, 1:1,000), anti‐MMP2 (Abcam, ab97779, 1:1,500), anti‐MMP9 (Abcam, ab38898, 1:1,500), and anti‐GAPDH (Abcam, ab9485, 1:2,000) at 4 °C overnight. The secondary antibodies (HRP goat anti‐rabbit, Abcam, ab205718, 1:2,000) were applied to incubate the membranes at 37 °C for 120 Min. The protein was exposed using ECL detection reagent.

### 2.9 Animal experiments

This experiments related with animals were performed according to the Institutional Animal Care and Use Committee and approved by Ethics Committee of Zhejiang Hospital. BALB/c nude mice (male, 6 weeks, 18–22 g, *n* = 12) were purchased from Guangdong Medical Laboratory Animal Center. The A549 cells transfected with EZH2 knockdown plasmid or control plasmid were adjusted at a density of 1 × 10^7^ cells/mL and were, respectively, injected into armpit of the nude mice subcutaneously (*n* = 6 per group). After 5 weeks, the mice were sacrificed by rapid cervical dislocation and then the xenograft tumor tissues were collected.

### 2.10 Hematoxylin and eosin staining and immunohistochemical assay

The tumor tissues were fixed with 4% formaldehyde at 4 ˚C overnight and then embedded with paraffin. The tissues were sectioned into 4–5 µm slices. For hematoxylin and eosin (HE) staining, the dewaxed sections were stained with hematoxylin (Sigma, St. Louis, MO, USA) for 15 Min, and then incubated in hydrochloric acid alcohol (1%) for 15 Sec. After soaking in eosin (Sigma) for 2 Min, the slices were observed under an optical microscope. For theimmunohistochemical (IHC) assay, the sections were stained with F4/80 macrophage marker, EZH2, CCL5, or MMP2 (1:100, ab100790; Abcam, USA). In brief, after deparaffinized with xylene, the sections were subject to retrieval in a heating citrate buffer (pH 6.0) for 15 Min. After cooling down to temperature, the sections were blocked with normal goat serum (Thermofisher) at 37 ˚C for 10 Min and then incubated with primary anti‐F4/80, EZH2, CCL5, or MMP2 antibodies overnight at 4 °C. Next, sections were incubated with anti‐rabbit secondary antibody (biotinylated) (1:1,000, ab6720, Abcam) at room temperature for 30 Min. The horseradish peroxidase‐conjugated streptavidin (Amersham, Amersham, UK) was incubated with sections at 37 ˚C for 30 Min. The diaminobenzidine (Sigma) was used for chromogenic detection. The staining was observed under an inverted microscope (Olympus, Tokyo, Japan) (400× magnification).

### 2.11 Statistical analysis

One‐way analysis of variance (ANOVA) with Turkey's test was applied to compare the difference among multiple groups (> 2 groups). Student's *t* text was used to compare the difference between groups. The data are shown as mean ± standard deviation. Statistical analyses were performed using GraphPad Prism 6.0 (GraphPad Software, La Jolla, CA, USA). *P* < 0.05 was considered to indicate a statistically significant difference.

## 3 Results

### 3.1 Downregulation of EZH2 in lung cancer cells inhibited chemotaxis of macrophages and decreased CCL5 expression

**FIG. 1:**
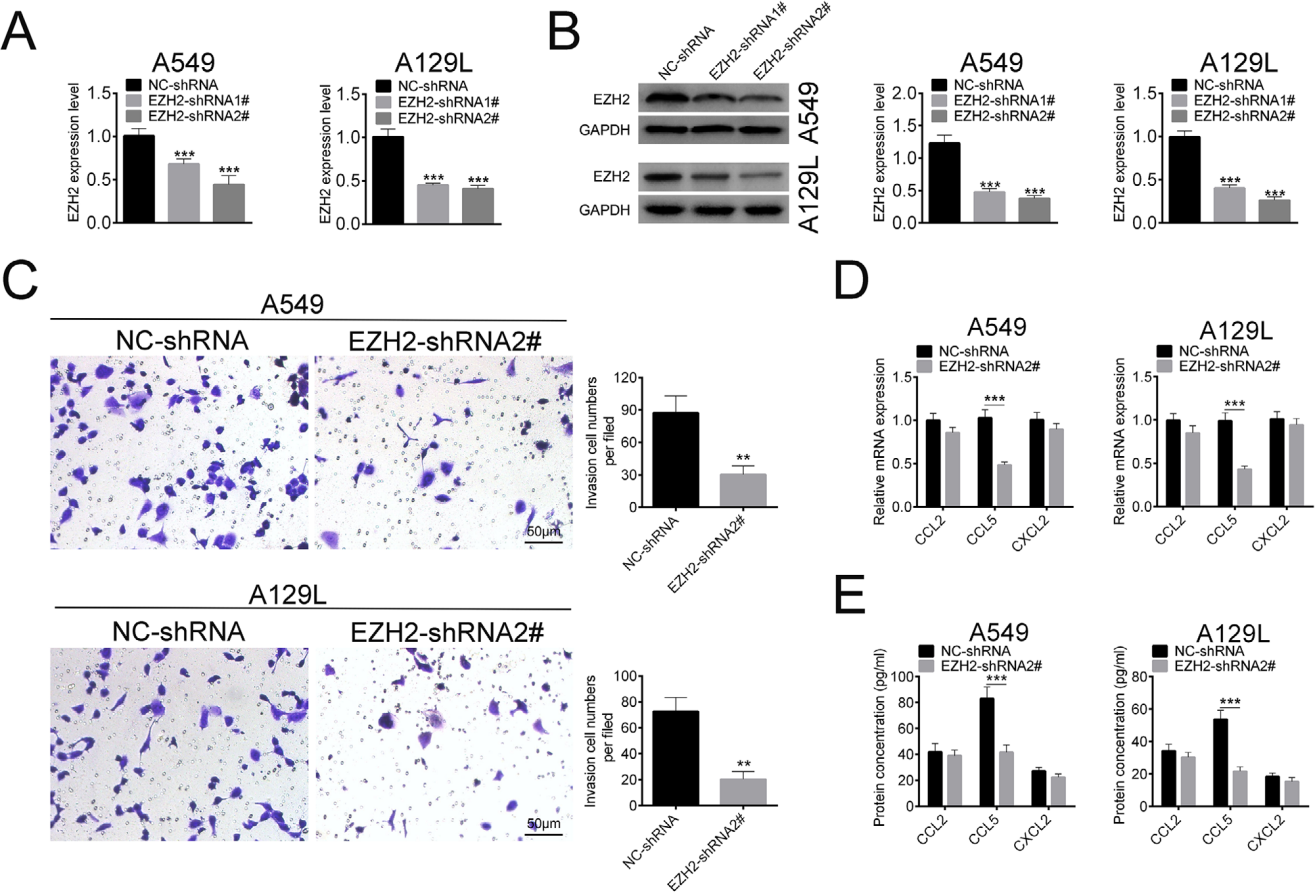
EZH2 knockdown in lung cancer cells inhibited chemotaxis of macrophages and decreased CCL5 expression. (A) Transfection efficiency of EZH2‐shRNA1# or 2# in A549 and A129L cells detected by qRT‐PCR. (B) Transfection efficiency of EZH2‐shRNA1# or 2# in A549 and A129L cells detected by Western blot. (C) Effect of EZH2‐shRNA2# on chemotaxis of macrophages was determined by transwell assay (400×). (D) Effect of EZH2‐shRNA2# on mRNA expression of CCL2, CCL5, and CXCL2 were determined by qRT‐PCR. (E) Effect of EZH2‐shRNA2# on protein expression of CCL2, CCL5, and CXCL2 were determined by ELISA. ***P < 0.001.

Since macrophage infiltration is reported to be associated with poor prognosis in lung cancer, we determined to detect the effect of EZH2 on macrophage infiltration of lung cancer. Human lung cancer cell lines A549 and A129L were first transfected with shRNAs for knocking down of EZH2, as shown by qRT‐PCR (Fig. 1A) and Western blot analysis (Fig. 1B). Transwell assay was then performed to detect the effect of EZH2 on the chemotaxis of macrophages. The results showed that the number of chemotactic migrated macrophages was significantly decreased when EZH2 was silenced in lung cancer cells (Fig. 1C), indicating that EZH2 knockdown inhibited the chemotaxis of macrophages. The inflammatory chemokine (CCL5) contributing to metastasis of cancer cells was downregulated in A549 and A129L transfected with EZH2‐shRNA2# in both mRNA (Fig. 1D) and protein (Fig. 1E) levels. However, the expression of CCL2 and CXCL2 remained stable in the cells transfected with EZH2‐shRNA2# (Figs. 1D and 1E). Taken together, EZH2 knockdown in lung cancer cells inhibited chemotaxis of macrophages and decreased CCL5 expression.

### 3.2 Downregulation of EZH2 inhibited migration and invasion of lung cancer cells

**FIG. 2:**
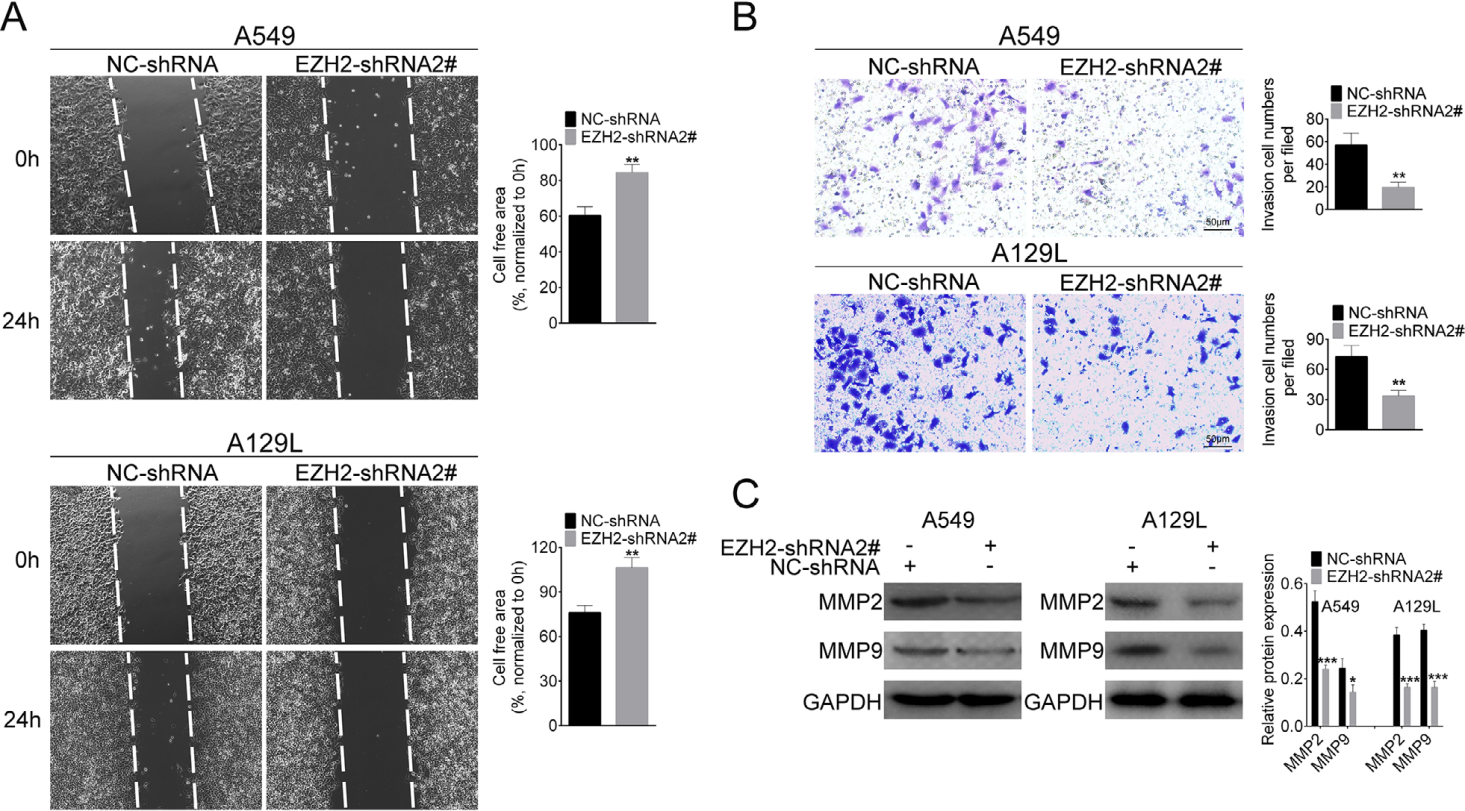
EZH2 knockdown inhibited migration and invasion of lung cancer cells. (A) Effect of EZH2‐shRNA2# on cell migration was determined by wound‐healing assay. (B) Effect of EZH2‐shRNA2# on cell invasion was determined by transwell assay. (C) Effect of EZH2‐shRNA2# on protein expression of MMP2 and MMP9 was determined by Western blot. *P < 0.05, **P < 0.01, ***P < 0.001.

The effects of EZH2 on cell migration and invasion of lung cancer cells were determined via loss‐of functional assays. First, the results from wound‐healing assay showed that knockdown of EZH2 markedly decreased cell migration of A549 and A129L (Fig. 2A). Second, transwell assay indicated that invasion ability of A549 and A129L cells was inhibited by EZH2 deletion (Fig. 2B). Matrix‐metalloproteinases (MMPs) (MMP2 and MMP9), closely related to the tumor cell metastasis, were downregulated by EZH2 knockdown (Fig. 2C). Taken together, these results indicated that knockdown of EZH2 inhibited migration and invasion of lung cancer cells.

### 3.3 EZH2 enhanced the recruitment of macrophages via upregulation of CCL5

**FIG. 3:**
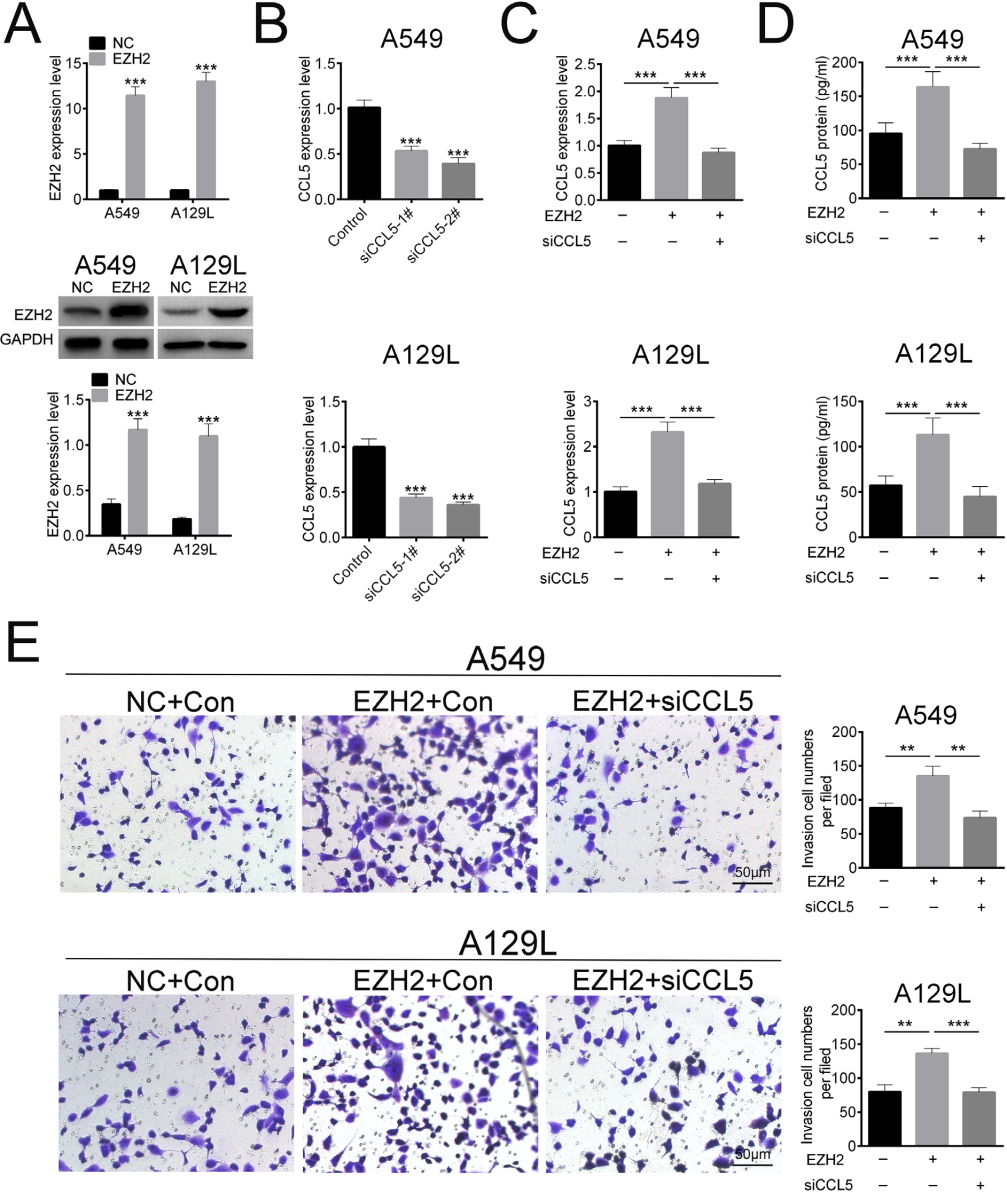
EZH2 promoted chemotaxis of macrophages via upregulation of CCL5. (A) Transfection efficiency of pcDNA3.1‐EZH2 was determined by qRT‐PCR and Western blot. (B) Transfection efficiency of si‐CCL5‐1# or 2# was determined by qRT‐PCR. (C) Effect of EZH2 and siCCL5 on mRNA expression of CCL5 was determined by qRT‐PCR. (D) Effect of EZH2 and siCCL5 on protein expression of CCL5 was determined by ELISA. (E) Effect of EZH2 and siCCL5 on chemotaxis of macrophages was determined by transwell. **P < 0.01, ***P < 0.001.

We next explored the role of CCL5 in the chemotaxis of macrophages. The efficiency of pcDNA3.1‐EZH2 (Fig. 3A) and siCCL5‐1# or 2# (Fig. 3B) was first determined. Meanwhile, siCCL5‐2# with the higher knockdown efficiency was selected for the subsequently experiments and named as siCCL5. The mRNA (Fig. 3C) and protein (Fig. 3D) expression of CCL5 were upregulated in A549 and A129L cells transfected with pcDNA3.1‐EZH2, whereas downregulated in cells cotransfected with pcDNA3.1‐EZH2 and siCCL5, suggesting that EZH2 could regulate CCL5 expression. Then, we performed macrophages chemotaxis‐transwell assay to detect the effect of CCL5 on chemotaxis of macrophages. The results showed that the number of chemotactic‐migrated macrophages was increased when EZH2 was overexpressed in lung cancer cells (Fig. 3E), indicating that EZH2 enhanced the chemotaxis of macrophages. However, addition of CCL5 downregulation reversed the effect induced by EZH2 overexpression, leading to the reduced migration of macrophages (Fig. 3E). These results suggested that EZH2 promoted chemotaxis of macrophages via upregulation of CCL5.

### 3.4 EZH2 promoted bioactivity of lung cancer cells via upregulation of CCL5

**FIG. 4:**
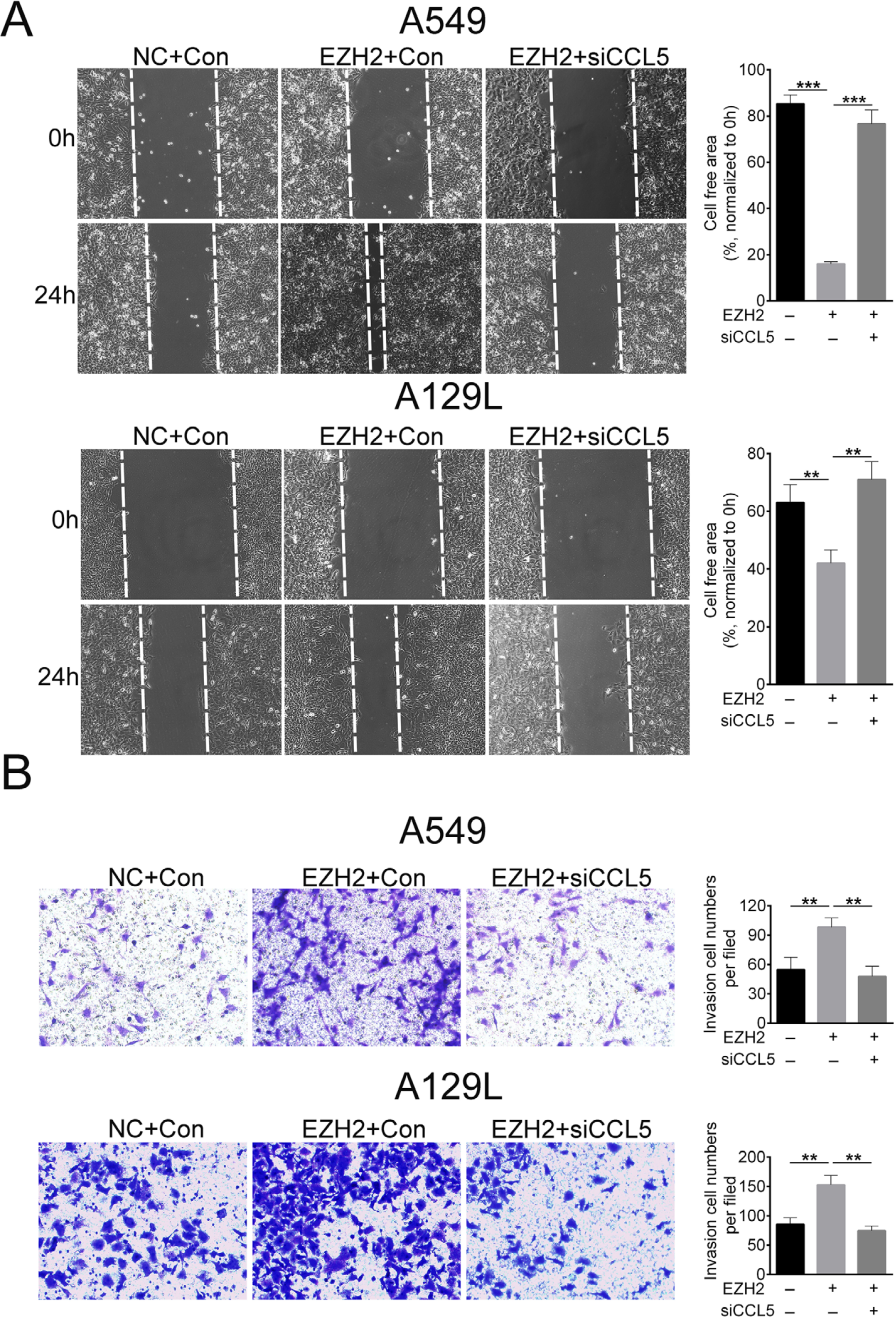
EZH2 promoted migration and invasion of lung cancer cells via upregulation of CCL5. (A) Effect of EZH2 and siCCL5 on cell migration was determined by wound‐healing assay. (B) Effect of EZH2 and siCCL5 on cell invasion was determined by transwell assay. ***P < 0.001.

Then, we explored the role of CCL5 in EZH2‐mediated migration and invasion of lung cancer cells and performed cell wound‐healing and transwell assays. The results displayed that the migration (Fig. 4A) and invasion (Fig. 4B) were promoted by EZH2 overexpression. In contrast, the cell migration and invasion was suppressed in EZH2+siCCL5 groups compared to EZH2+Con groups in A549 and A129L cells (Figs. 4A and 4B). These results indicated that EZH2 promoted migration and invasion of lung cancer cells via upregulation of CCL5.

### 3.5 Downregulation of EZH2 inhibited the *in vivo* macrophages infiltration of lung cancer

**FIG. 5:**
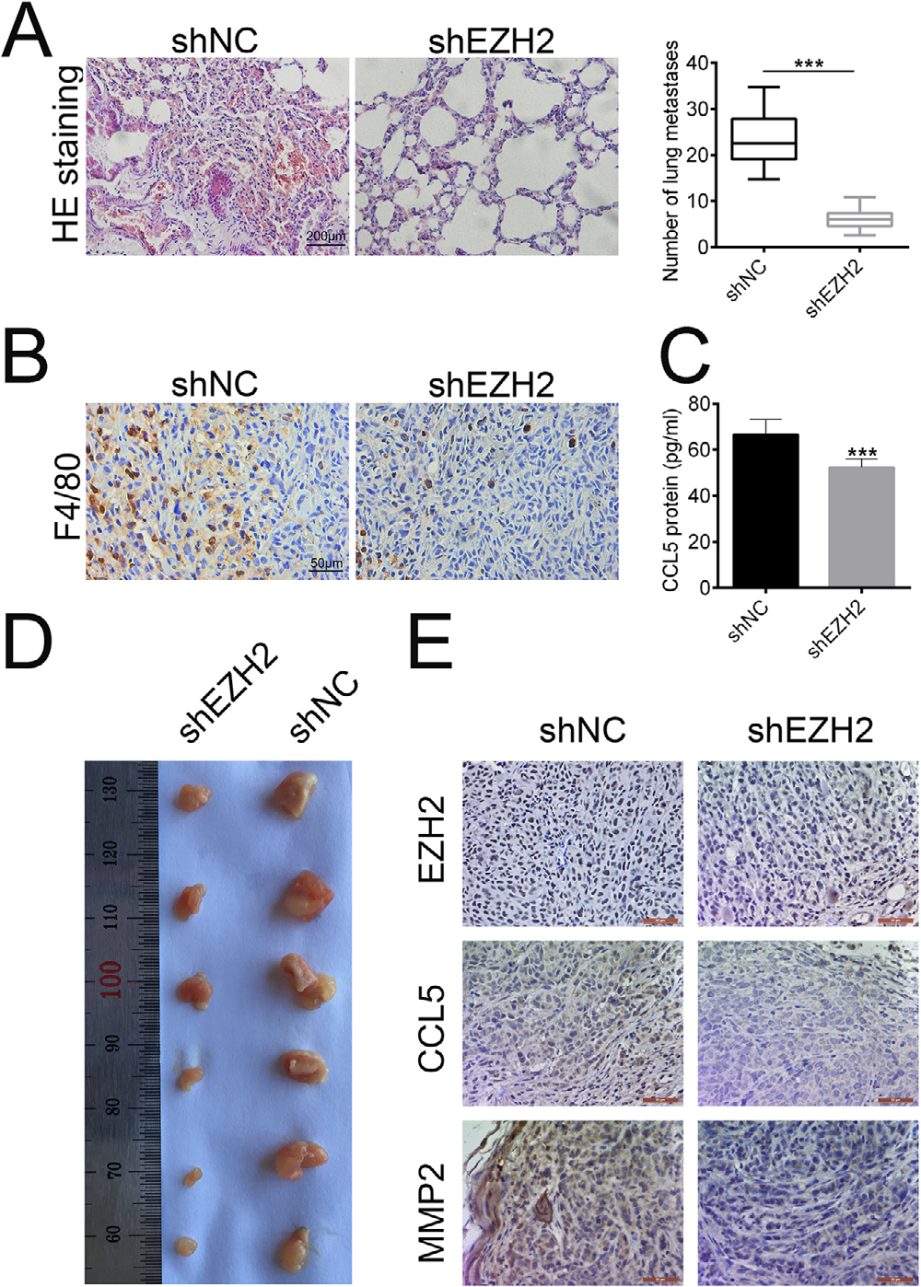
EZH2 knockdown inhibited the *in vivo* macrophages infiltration of lung cancer. (A) The lung cancer metastasis influenced by shEZH2 was observed by HE staining. Scale bar: 200 µm. (B) The expression of F4/80 influenced by shEZH2 was measured by IHC assay. Scale bar: 50 µm. (C) Protein secretion of CCL5 influenced by shEZH2 was evaluated by ELISA. ***P < 0.001. (D) Representative images of tumor tissues influenced by shEZH2. (E) The expression of EZH2, CCL5, and MMP2 influenced by shEZH2 was measured by IHC assay. Scale bar: 200 µm.

To further validate the clinical significance of EZH2 on TME of lung cancer cells, we determined the lung metastasis through HE staining. The data showed that knockdown of EZH2 significantly reduced the number of lung metastasis (Fig. 5A). The expression of macrophage biomarker, F4/80‐a, was decreased by the knockdown of EZH2 (Fig. 5B), indicating that the infiltration of macrophages was reduced by EZH2 knockdown. In addition, results from ELISA showed that the expression of CCL5 in lung cancer tissues was decreased by EZH2 knockdown (Fig. 5C). Moreover, knockdown of EZH2 inhibited lung tumor growth (Fig. 5D). IHC staining showed decreased expression of EZH2, CCL5, and MMP2 in tumor tissues (Fig. 5E). Taken together, the lung metastasis and infiltration of macrophages were inhibited by EZH2 knockdown, in which progress the downregulation of CCL5 was closely involved.

## 4 Discussion

It has demonstrated that the survival, growth, proliferation, and metastasis of tumor cells are greatly influenced by the signal communication between tumor cells and TME, especially in lung cancer 20. EZH2 is capable of regulating immune cells, including T cells and macrophages, thus performing decisive roles in regulation of TME and cell invasion in cancer cells 21. However, although EZH2 has widely known to be a critical regulator of lung cancer progression, the definite mechanism of EZH2 in macrophage recruitment of lung cancer was not fully elucidated. Thus, we aimed to validate the underlying mechanism of the effects of EZH2 on lung cancer.

In this study, we proved that knockdown of EZH2 inhibited the migration and invasion of lung cells, consistent with previous studies 22, 23. MMPs play critical roles in the homeostatic modulation of the extracellular environment and are closely related to the poor clinical prognosis 24. MMP2/9, belonging to the MMPs family, has been shown to be associated with poor prognosis and metastasis in lung cancer 25. The present study indicated that EZH2 knockdown decreasedthe expression of MMP2/9, suggesting that EZH2 knockdown exerted tumor suppressor role in lung cancer cells.

Other than MMPs, tumor‐associated macrophages could also regulate the metastasis of tumor cells and high infiltration of macrophages may imply the high risk of distant metastasis and poor prognosis 26 of cancer, including lung cancer 27. The downregulation of EZH2 decreases cytokine secretion and inhibits macrophage‐dependent disease development 28. Thus, we speculated that the infiltration of macrophages may be modulated by EZH2. Transwell assay in the present study showed that the number of migrated macrophages was decreased when EZH2 was downregulated in lung cancer cells. By contrast, when EZH2 was upregulated in lung cancer cells, the number of migrated macrophages was increased. Therefore, these findings suggested that EZH2 knockdown inhibited the macrophage recruitment in lung cancer cells.

Chemokines, as small secreted molecules, are able to regulate the migration of several cell types 29 and related to macrophage recruitment 30. CCL5 could increase lung cancer migration 31 and induce macrophage infiltration 32, thus promoting lung cancer progression. The expression of CCL5 in this study was downregulated by EZH2 knockdown, while upregulated by EZH2 overexpression, suggesting that CLL5 is bona fide target of EZH2, which is also confirmed by Yang et al. 33. Moreover, a previous study has shown that EZH2 plays anti‐inflammatory activity, as shown by a decrease of CCL5 via EZH2 inhibition 34. However, CCL5 was upregulated in long‐term hematopoietic stem cells with EZH2 deletion 33. EZH2 contains activating or inactivating mutations in various tumors, and thus playing oncogenic or tumor suppressor functions in human malignancies 33. Therefore, different mutations of EZH2 may result in pro‐ or anti‐inflammatory activities. Furthermore, knockdown of CCL5 inhibited the macrophage recruitment, cell migration, and invasion, which were promoted by EZH2 overexpression. These results indicated that EZH2 overexpression promoted the macrophage recruitment, cell migration, and invasion via upregulating the expression of CCL5 in lung cancer cells. The *in vivo* subcutaneous xenotransplanted tumor model further confirmed that EZH2 knockdown inhibited lung cancer metastasis and macrophage recruitment, indicated by downregulation of molecular macrophage marker, F4/80 35. Meanwhile, the protein secretion of CCL5 was also inhibited by EZH2. Altogether, these results demonstrated that EZH2 knockdown inhibited lung cancer metastasis and macrophage infiltration, and the reduced expression of CCL5 may be a partial reason for these phenomena. Notably, it is interesting to further investigate how EZH2 affects the expression of CCL5 in future studies to explore the precise interference mechanism for CCL5 in preventing lung cancer. In addition, whether CCL5 deletion can reverse the effects of Raf kinase inhibitor protein (RKIP) remains to be elucidated *in vivo*. Moreover, although CCL2 36 and CXCL2 37 have been shown to have relations with lung cancer metastasis and macrophage recruitment, the expression of CCL2 and CXCL2 was not influenced by EZH2 in the present study. The different regulation on CCL2 and CXCL2 may be caused by different cell context and study model.

## 5 Conclusion

In summary, the current study demonstrated that EZH2 acted as a tumor promoter in lung cancer. Knockdown of EZH2 reduced the CCL5 protein secretion and therefore inhibited the macrophage recruitment, finally leading to the decreased invasion and metastasis ability of lung cancer cells. Our study may provide candidate molecular target and potential treatment strategies in preventing lung cancer.

## References

[1] Bray, F. , Ferlay, J. , Soerjomataram, I. , Siegel, R. L. , Torre, L. A. , and Jemal, A . (2018) CA Cancer J. Clin. 68(6), 394–424. 30207593 10.3322/caac.21492

[2] Hoffman, P. C. , Mauer, A. M. , and Vokes, E. E . (2000) Lancet. 355(9202), 479–485. 10841143 10.1016/S0140-6736(00)82038-3

[3] Pao, W. , Girard, N. (2011) Lancet Oncol. 12(2), 175–180. 21277552 10.1016/S1470-2045(10)70087-5

[4] Tan, H. ‐ Y. , Wang, N. , Lam, W. , Guo, W. , Feng, Y. , and Cheng, Y. ‐ C. (2018) Mol. Cancer. 17(1), 43. 29455663 10.1186/s12943-018-0800-6 PMC5817793

[5] Jin, K. , Pandey, N. B. , and Popel, A. S . (2017) Oncotarget. 8(36), 60210–60222. 28947965 10.18632/oncotarget.19417 PMC5601133

[6] Qian, B. ‐ Z. , and Pollard, J. W. (2010) Cell. 141(1), 39–51. 20371344 10.1016/j.cell.2010.03.014 PMC4994190

[7] Mei, J. , Xiao, Z. , Guo, C. , Pu, Q. , Ma, L. , Liu, C. , Lin, F. , Liao, H. , You, Z. , and Liu, L. (2016) Oncotarget. 7(23), 34217–34228. 27144518 10.18632/oncotarget.9079 PMC5085150

[8] Bonecchi, R. , Locati, M. , and Mantovani, A . (2011) Cancer Cell. 19(4), 434–435. 21481784 10.1016/j.ccr.2011.03.017

[9] Brauß, T. F. , Winslow, S. , Lampe, S. , Scholz, A. , Weigert, A. , Dehne, N. , von Stedingk, K. , Schmid, T. , and Brüne, B. (2017) Mol. Carcinog. 56(12), 2620–2629. 28731284 10.1002/mc.22706

[10] Borsig, L. , Wolf, M. J. , Roblek, M. , Lorentzen, A. , and Heikenwalder, M. (2014) Oncogene. 33(25), 3217. 23851506 10.1038/onc.2013.272

[11] Panni, R. Z. , Linehan, D. C. , and DeNardo, D. G . (2013) Immunotherapy. 5(10), 1075–1087. 24088077 10.2217/imt.13.102 PMC3867255

[12] Marchesi, I. , and Bagella, L. (2016) World J. Clin. Oncol. 7(2), 135. 27081636 10.5306/wjco.v7.i2.135 PMC4826959

[13] Wen, Y. , Cai, J. , Hou, Y. , Huang, Z. , and Wang, Z. (2017) Oncotarget. 8(23), 37974–37990. 28415635 10.18632/oncotarget.16467 PMC5514966

[14] Kim, K. H. , and Roberts, C. W. M. (2016) Nat. Med. 22(2), 128–134. 26845405 10.1038/nm.4036 PMC4918227

[15] Hussain, M. , Rao, M. , Humphries, A. E. , Hong, J. A. , Liu, F. , Yang, M. , Caragacianu, D. , and Schrump, D. S. (2009) Cancer Res. 69(8), 3570–3578. 19351856 10.1158/0008-5472.CAN-08-2807 PMC8374472

[16] Liu, H. , Li, W. , Yu, X. , Gao, F. , Duan, Z. , Ma, X. , Tan, S. , Yuan, Y. , Liu, L. , Wang, J. , Zhou, X. , Yang, Y . (2016) Oncotarget. 7(35), 56338–56354. 10.18632/oncotarget.10841 27472460 PMC5302918

[17] Murai, F. , Koinuma, D. , Shinozaki‐Ushiku, A. , Fukayama, M. , Miyaozono, K. , and Ehata, S. (2015) Cell Discovery. 1, 15026. 27462425 10.1038/celldisc.2015.26 PMC4860843

[18] Zhang, C. , Liu, T. , Wang, G. , Wang, H. , Che, X. , Gao, X. , and Liu, H. (2017). J. Cancer. 8(13), 2511. 28900489 10.7150/jca.18161 PMC5595081

[19] Cheng, Y. , Pan, Y. , Pan, Y. , and Wang, O. (2019) Cancer Manage. Res. 11, 803. 10.2147/CMAR.S188007 PMC6340505 30697072

[20] Wood, S. L. , Pernemalm, M. , Crosbie, P. A. , and Whetton, A. D. (2014) Cancer Treat. Rev. 40(4), 558–566. 24176790 10.1016/j.ctrv.2013.10.001

[21] Gan, L. , Yang, Y. , Li, Q. , Feng, Y. , Liu, T. , and Guo, W. (2018) Biomark. Res. 6, 10. 29556394 10.1186/s40364-018-0122-2 PMC5845366

[22] Frankel, A. E. , Liu, X. , and Minna, J. D . (2016) Cancer Discov. 6(9), 949–952. 27587466 10.1158/2159-8290.CD-16-0800 PMC5012289

[23] Zhang, H. , Qi, J. , Reyes, J. M. , Li, L. , Rao, P. K. , Li, F. , Lin, C. Y. , Perry, J. A. , Lawlor, M. A. , Federation, A. , De Raedt, T. , Li, Y. Y. , Liu, Y. , Duarte, M. A. , Zhang, Y. , Herter‐Sprie, G. S. , Kikuchi, E. , Carretero, J. , Perou, C. M. , Reibel, J. B. , Paulk, J. , Bronson, R. T. , Watanabe, H. , Brainson, C. F. , Kim, C. F. , Hammerman, P. S. , Brown, M. , Cichowski, K. , Long, H. , Bradner, J. E. , and Wong, K. ‐ K. (2016) Cancer Discov. 6(9), 1006–1021. 27312177 10.1158/2159-8290.CD-16-0164 PMC5010480

[24] Overall, C. M. , and Dean, R. A. (2006) Cancer Metastasis Rev. 25(1), 69–75. 16680573 10.1007/s10555-006-7890-0

[25] Vandenbroucke, R. E. , Dejonckheere, E. , and Libert, C . (2011) Eur. Respir. J. 38(5), 1200–12014. 21659416 10.1183/09031936.00027411

[26] Yuan, Z. ‐ Y. , Luo, R. ‐ Z. , Peng, R. ‐ J. , Wang, S. ‐ S. , and Xue, C. (2014) OncoTargets Ther. 7, 1475–1480. 10.2147/OTT.S61838 PMC4149399 25187727

[27] Ma, J. , Liu, L. , Che, G. , Yu, N. , Dai, F. , and You, Z. (2010) BMC Cancer. 10, 112. 20338029 10.1186/1471-2407-10-112 PMC2851690

[28] Neele, A. E. , and de Winther, M. P.J . (2018) J. Exp. Med. 215(5), 1269–1271. 29691302 10.1084/jem.20180479 PMC5940273

[29] Young, M. R. (2000) Arch. Pathol. Lab. Med. 124(4), 642. 10747331

[30] De Filippo, K. , Dudeck, A. , Hasenberg, M. , Nye, E. , van Rooijen, N. , Hartmann, K. , Gunzer, M. , Roers, A. , and Hogg, N. (2013) Blood. 121(24), 4930–4937. 23645836 10.1182/blood-2013-02-486217

[31] Huang, C. ‐ Y. , Fong, Y. ‐ C. , Lee, C. ‐ Y. , Chen, M. ‐ Y. , Tsai, H. ‐ C. , Hsu, H. ‐ C. , and Tang, C. ‐ H. (2009) Biochem. Pharmacol. 77(5), 794–803. 19073147 10.1016/j.bcp.2008.11.014

[32] Wang, X. , Yang, X. , Tsai, Y. , Yang, L. , Chuang, K. ‐ H. , Keng, P. C. , Lee, S. O. , and Chen, Y. (2017) Radiat. Res. 187(1), 50–59. 28054838 10.1667/RR14503.1

[33] Yang, Y. , Akada, H. , Nath, D. , Hutchison, R. E. , and Mohi, G. (2016) Blood. 127(26), 3410–3423. 27081096 10.1182/blood-2015-11-679431 PMC4929929

[34] Qi, W. , Chan, H. , Teng, L. , Li, L. , Chuai, S. , Zhang, R. , Zeng, J. , Li, M. , Fan, H. , Lin, Y. , Gu, J. , Ardayfio, O. , Zhang, J. ‐ H. , Yan, X. , Fang, J. , Mi, Y. , Zhang, M. , Zhou, T. , Feng, G. , Chen, Z. , Li, G. , Yang, T. , Zhao, K. , Liu, X. , Yu, Z. , Lu, C. X. , Atadja, P. , and Li, E. (2012) Proc. Natl. Acad. Sci. 109(52), 21360–21365. 23236167 10.1073/pnas.1210371110 PMC3535655

[35] Dos Anjos Cassado, A. (2017) Results Probl. Cell Differ. 62, 161–179. 28455709 10.1007/978-3-319-54090-0_7

[36] Lim, S. Y. , Yuzhalin, A. E. , Gordon‐Weeks, A. N. , and Muschel, R. J. (2016) Oncotarget. 7(19), 28697–28710. 26885690 10.18632/oncotarget.7376 PMC5053756

[37] Saintigny, P. , Massarelli, E. , Lin, S. , Ahn, Y. ‐ H. , Chen, Y. , Goswami, S. , Erez, B. , O'Reilly, M. S. , Liu, D. , Lee, J. J. , Zhang, L. , Ping, Y. , Behrens, C. , Solis Soto, L. M. , Heymach, J. V. , Kim, E. S. , Herbst, R. S. , Lippman, S. M. , Wistuba, I. I. , Hong, W. K. , Kurie, J. M. , and Koo, J. S. (2013) Cancer Res. 73(2), 571‐82. 23204236 10.1158/0008-5472.CAN-12-0263 PMC3548940

